# Comparing the Effectiveness of Artificial Intelligence Models in Predicting Ovarian Cancer Survival: A Systematic Review

**DOI:** 10.1002/cnr2.70138

**Published:** 2025-03-19

**Authors:** Farkhondeh Asadi, Milad Rahimi, Nahid Ramezanghorbani, Sohrab Almasi

**Affiliations:** ^1^ Department of Health Information Technology and Management, School of Allied Medical Sciences Shahid Beheshti University of Medical Sciences Tehran Iran; ^2^ Department of Development & Coordination Scientific Information and Publications Deputy of Research & Technology, Ministry of Health & Medical Education Tehran Iran

**Keywords:** artificial intelligence, cancer, machine learning, ovary, survival

## Abstract

**Background:**

This systematic review investigates the use of machine learning (ML) algorithms in predicting survival outcomes for ovarian cancer (OC) patients. Key prognostic endpoints, including overall survival (OS), recurrence‐free survival (RFS), progression‐free survival (PFS), and treatment response prediction (TRP), are examined to evaluate the effectiveness of these algorithms and identify significant features that influence predictive accuracy.

**Recent Findings:**

A thorough search of four major databases—PubMed, Scopus, Web of Science, and Cochrane—resulted in 2400 articles published within the last decade, with 32 studies meeting the inclusion criteria. Notably, most publications emerged after 2021. Commonly used algorithms for survival prediction included random forest, support vector machines, logistic regression, XGBoost, and various deep learning models. Evaluation metrics such as area under the curve (AUC) (18 studies), concordance index (C‐index) (11 studies), and accuracy (11 studies) were frequently employed. Age at diagnosis, tumor stage, CA‐125 levels, and treatment‐related factors were consistently highlighted as significant predictors, emphasizing their relevance in OC prognosis.

**Conclusion:**

ML models demonstrate considerable potential for predicting OC survival outcomes; however, challenges persist regarding model accuracy and interpretability. Incorporating diverse data types—such as clinical, imaging, and molecular datasets—holds promise for enhancing predictive capabilities. Future advancements will depend on integrating heterogeneous data sources with multimodal ML approaches, which are crucial for improving prognostic precision in OC.

**Abbreviations**
ABDSN: attention‐based deep survival network
AUC: area under the curve
CLAM: clustering‐constrained‐attention multiple‐instance learning
COX‐PH: Cox proportional hazards
CPH: Cox proportional hazards
CT: computed tomographyn
DL: deep learning
DL‐CPH: deep learning Cox proportional hazards
DT: decision tree
EL: ensemble learning
GA‐XG: Boost Genetic Algorithm XGBoost
GB: gradient boosting
GEO: Gene Expression Omnibus
GNN: graph neural network
KNN: *K*‐nearest neighbors
LR: logistic regression
ML: machine learning
MLDP: machine learning–derived prognostic signature
MRI: magnetic resonance imaging
NPV: negative predictive value
OS: overall survival
PFS: progression‐free survival
PLS: partial least squares
PPV: positive predictive value
PRISMA: Preferred Reporting Items for Systematic Reviews and Meta‐Analyses
RF: random forest
RFS: recurrence‐free survival
SEER: Surveillance, Epidemiology, and End Results
SMC: Samsung Medical Center
SVM: support vector machines
TCGA: The Cancer Genome Atlas
TRP: treatment response prediction
ViT‐DL: vision transformer–based deep learning
WSI: whole slide image

## 1 Introduction

Ovarian cancer (OC) is a significant global health concern, ranking seventh in prevalence and eighth in mortality among women worldwide [1]. It is the third leading cause of death among gynecological cancers, following uterine and cervical cancers, with over 300 000 cases diagnosed annually and nearly 200 000 deaths. Despite advancements in diagnosis and treatment, OC remains a formidable challenge due to its often asymptomatic nature in early stages, leading to late‐stage diagnoses and poor prognosis [2]. The global incidence of OC is estimated at 6.6 per 100 000 women per year, and it has been the fifth most common cause of cancer‐related death in women in the Western world over the last two decades [3].

OC tumors are classified into three categories as follows: benign, borderline, and malignant [4]. While malignant neoplasms often necessitate invasive surgical interventions, benign masses can be managed with safer monitoring approaches. The poor prognosis associated with OC is largely due to the advanced metastatic stages at which many patients are diagnosed [5]. Variations in OC incidence and mortality rates across different regions emphasize the need to understand the diverse epidemiology influenced by factors such as age, reproductive history, hormonal imbalances, genetic predispositions, and lifestyle choices [6]. A family history of OC, particularly involving first‐degree relatives, significantly increases an individual's risk, often due to hereditary mutations in the *BRCA1* and *BRCA2* genes [7].

Despite substantial progress in treatment options, the overall survival (OS) rate for OC patients has seen only modest improvements. The GLOBOCAN project estimates that by 2050, the global incidence of OC will rise by 55%, with the highest burden expected to fall on low‐ and middle‐income countries (LMICs) [2]. Approximately 75% of cases occur in postmenopausal individuals, with an incidence rate of 40 per 100 000 annually in those aged 50 and above. Early detection significantly boosts the 5‐year survival rate from 3% at Stage IV to 90% at Stage I [8]. Key factors influencing survival include cancer stage, tumor size, residual tumor after surgery, tissue type, and race [9]. The limited improvement in survival rates is partly attributable to the lack of effective prognostic biomarkers and screening methods, leading to delayed diagnoses [6].

Accurate prognosis and survival predictions are critical in managing OC, aiding in treatment decisions and reducing patient anxiety [10]. However, oncologists often overestimate survival, leading to inadequate end‐of‐life care. Studies, such as those by Alexi et al., have shown that oncologists' prognoses can be overly optimistic, especially in long‐term patient relationships, as is common in OC cases [11]. This highlights the need for reliable prognostic tools that can assist in clinical decision‐making, particularly in identifying patients nearing the end of life.

Traditional statistical methods have been widely used to predict cancer prognosis, including in OC [12, 13, 14]. For instance, the multivariable Cox proportional hazards (CPH) model has been employed to assess OS in OC patients [13]. However, the emergence of machine learning (ML) algorithms offers a promising alternative, providing enhanced accuracy and the ability to handle complex, nonlinear data patterns [15, 16]. ML, a subset of artificial intelligence, has demonstrated significant potential in survival analysis, particularly when combined with deep learning (DL) techniques. DL models, known for their layered architecture and capacity for automatic feature selection, have shown remarkable performance in various cancer survival predictions, including breast [17], cervical [18], lung [19], and bladder cancers [20].

Although ML and DL have shown advantages over traditional statistical models in various survival analyses, their potential for enhancing OC survival prediction remains to be fully explored [21]. This review aims to systematically analyze all original studies that have utilized ML algorithms to predict OC survival. The focus will be on evaluating models based on OS, recurrence‐free survival (RFS), progression‐free survival (PFS), and treatment response prediction (TRP). The review will assess each step of the modeling process, from data collection to performance evaluation, and will identify key variables influencing survival predictions, categorizing them based on survival type.

## 2 Methods

This systematic review was meticulously conducted in alignment with the Preferred Reporting Items for Systematic Reviews and Meta‐Analyses (PRISMA) guidelines, ensuring a comprehensive and standardized approach to data collection and analysis [22].

### 2.1 Information Sources

A literature search was systematically conducted across multiple databases, including PubMed, Scopus, Web of Science, and Cochrane Library. Additionally, reference lists of relevant studies were examined to ensure comprehensive coverage of the literature. The final search across all sources was completed in December 2024.

### 2.2 Search Strategy and Selection Process

The search strategy aimed to identify relevant studies in three primary domains: OC, ML, and survival prediction. A combination of controlled vocabulary (MESH terms) and keywords was employed to construct the search queries, details of which are provided in Supplementary file. A 10‐year date filter was applied to focus on the most recent research. The selection process involved an independent screening of titles and abstracts, and full texts by two authors to assess their eligibility. Discrepancies were addressed through consultation with a corresponding author, ensuring consistency and reducing bias. No automation tools were utilized during the selection process.

### 2.3 Eligibility Criteria

Inclusion and exclusion criteria were established prior to the review. Studies were included if they were original research articles published in English, employed ML models to predict OC survival, clearly presented the model's output and evaluation, and specifically focused on OC. Exclusion criteria encompassed books, letters to the editor, review articles, and meta‐analyses, as well as studies that did not focus on OC or lacked detailed information regarding input datasets, modeling processes, and model evaluation (Table 1).

**TABLE 1:** Inclusion and exclusion criteria.

Inclusion criteria	Exclusion criteria
Only journals and original papers are used	Studies written in other languages
Studies written in English only	Other sources, such as books, theses, editorials, and systematic reviews
Papers that focused on ML– or DL–based ovarian cancer survival prediction	Papers that focused on statistical techniques for ovarian cancer survival prediction
	Studies without enough modeling details

### 2.4 Data Collection Process

Data extraction was conducted using a custom‐designed form tailored for this review. Two authors independently extracted relevant information from each study, including author details, publication year, country of origin, dataset characteristics, data types, ML models used, criteria for model performance assessment, and significant features influencing model outcomes. Data extraction focused on key outcomes, such as OS, PFS, RFS, and TRP. For studies reporting multiple outcomes or models, the data related to survival outcomes and the models demonstrating the best performance were prioritized (Table 2). Data synthesis involved summarizing the results through descriptive statistics and narrative synthesis.

**TABLE 2:** Characteristics of included articles.

Author, country, year	Year	Dataset	Participants/data records	Survival outcome	Best ML model	Results (eval)
Chih‐Jen T, Taiwan, (2017) [23]	2017	Chung Shan Medical University Hospital Tumor Registry	987	RFS	C5.0	Accuracy (0.90)
Paik ES, Korea, (2019) [24]	2019	Samsung Medical Center, Asan Medical Center	1128	OS	GB	AUC (0.84)
Wang S, China, (2019) [25]	2019	Two hospitals	8917	RFS	DL‐CPH	C‐index (0.71)
Arezzo F, Italy, (2020) [26]	2020	Tertiary center (2018–2019)	64	PFS	RF	Accuracy (0.93)
						Sensitivity (0.90)
						Precision (0.90)
						AUC (0.92)
Kaur I, India, (2021) [27]	2021	Rajiv Gandhi Cancer Institute & Research Center	140	OS	EL	Accuracy (0.71)
						Sensitivity (0.79)
						Specificity (0.61)
						AUC (0.80)
Hsiao YW,Taiwan, (2021) [28]	2021	GEO	106	OS	GA‐XGBoost	Accuracy (0.58)
						Sensitivity (0.82)
						Specificity (0.38)
						F1‐score (0.65)
He T, India, (2021) [29]	2021	TCGA GEO	790	OS	RF‐LR‐COX	C‐index (0.76)
Li HM, China, (2021) [30]	2021	Fudan University Shanghai Cancer Center (2014–2019)	117	RFS	DL—SVM	C‐index (0.62)
						AUC (0.85)
Shannon NB, Singapore, (2021) [31]	2021	GDSC, TCGA,GEO	—	TRP	LR XGB	Accuracy (0.82)
Feng Y, China, (2022) [32]	2022	Hospital (2009–2018)	98	OS	DT	AUC (0.69)
Sorayaie Azar A, Iran, (2022) [33]	2022	SEER	42 827	OS	RF	Accuracy (0.88)
						Sensitivity (0.71)
						Specificity (0.92)
						F1‐score (0.71)
						AUC (0.82)
Li Y, China, (2022) [34]	2022	TCGA,GEO	532	OS	XGBoost	AUC (0.72)
Zheng Y, China, (2022) [35]	2022	Qilu Hospital of Shandong University	734	OS	ViT‐DL	C‐index (0.74)
						AUC (0.82)
Chris S, USA, (2022) [36]	2022	Academic Cancer Institution	245	OS	EL	Accuracy (0.79)
						Sensitivity (0.71)
						Specificity(0.80)
						AUC (0.76)
Nero C, Italy, (2022) [37]	2022	Fondazione Policlinico Universitario (2016–2020)	664	PFS	DL	AUC (0.71)
						NPV (0.69)
						PPV (0.75)
Avesani G, Italy, (2022) [38]	2022	Multicentric database from four referral centers	218	PFS	XGBoost	AUC (0.62)
Tingyuan L, China, (2023) [39]	2023	TCGA	284	OS	DNN	AUC (0.94)
Meixuan W, China, (2023) [40]	2023	TCGA‐OV	90	OS	ABDSN	C‐index (0.58)
Qing H, China, (2023) [41]	2023	12 multicenter cohorts	2626	OS	MLDPS	C‐index (0.79)
Xiangmei L, China, (2023) [42]	2023	SEER (2004–2015)	1131	OS	LR	Accuracy (0.80)
						Precision (0.56)
						AUC (0.80)
Sheng W, China, (2023) [43]	2023	TCIA, TCGA,	489	OS	LASSO Regression	AUC (0.72)
David M, USA, (2023) [44]	2023	Academic institution	71	RFS	Elastic Net	C‐index (0.72)
Lili L, China, (2023) [45]	2023	Hospital of Chongqing Medical University (2013–2019)	3839	RFS	DL	C‐index (0.75)
						AUC (0.98)
Pradeep G, India, (2024) [46]	2024	OCD, SEER	9450	OS	Temporal GNN	Accuracy (0.95)
Lindong J, USA, (2024) [47]	2024	BRCA TCGA	355	OS	DL	C‐index (0.73)
Mirielle M, USA, (2024) [48]	2024	TCGA	360	OS	LR	Accuracy (0.64)
						Precision (0.68)
						Recall (0.34)
Yongmei H, USA, (2024) [49]	2024	SEER(2010–2015)	5656	OS	PLS Regression	C‐index (0.75)
Lian J, China, (2024) [50]	2024	Hospital (2013–2015)	102	PFS	RF	AUC (0.77)
Li‐Rong Y, China, (2024) [51]	2024	Yunnan Cancer Hospital (2012–2022)	1392	RFS	XGBoost	AUC (0.78)
						Sensitivity (0.73)
						Specificity (0.71)
						Accuracy (0.80)
Wan‐Chun L, Taiwan, (2024) [52]	2024	Two tertiary centers (2010–2019)	723	TRP	CatBoost	AUC (0.87)
						F1‐score (0.69)
Zijian Y, China, (2024) [53]	2024	TCGA‐OV	634	TRP	GNN	
Qiwang L, China, (2024) [54]	2024	TCGA, GEO	381	TRP	RSF	C‐index (0.75)

## 3 Results

**FIGURE 1:**
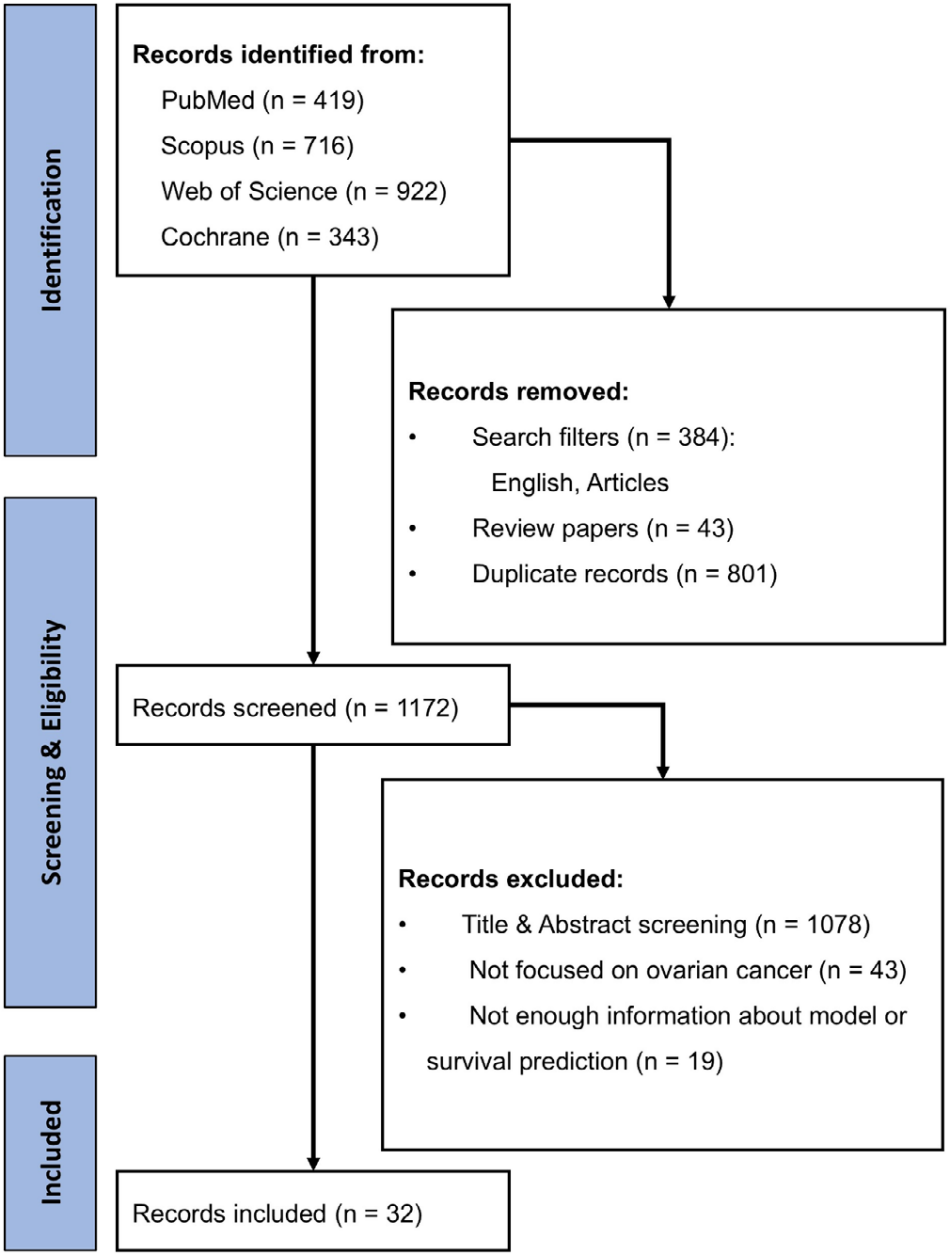
PRISMA diagram.

The initial search across the four specified databases yielded a total of 2400 articles. After applying filters to limit studies to those published within the last 10 years, excluding non‐original papers, and removing duplicates, 1172 articles remained. Subsequent screening resulted in the selection of 32 original articles that met the inclusion criteria for this systematic review (Figure 1).

### 3.1 Study Characteristics

The characteristics of the studies included in this review are summarized in Table 2. Most studies were published post‐2021, with a significant concentration of research originating from Asia (24 articles), followed by the United States (5 articles), and Europe (3 articles). OS was the most frequently predicted outcome, featured in 18 studies, whereas RFS was addressed in 16 studies. PFS and TRP were each the focus of four studies. A diverse range of ML and DL models was employed for survival prediction across the studies (Table 3).

**TABLE 3:** Categorizing the characteristics of the included studies.

Characteristics	Categories	OS (*n*)	PFS (*n*)	RFS (*n*)	TRP (*n*)
Geographic	Asia	14 (23, 27, 30–33, 44, 45, 47, 49–51, 71, 72)	1 (39)	5 (25, 28, 29, 40, 42)	4 (43, 48, 52, 53)
	Europe	—	3 (24, 37, 38)	—	—
	USA	4 (26, 34, 54, 55)	—	1 (41)	—
Public datasets	TCGA	7 (45, 47, 49, 51, 54, 55, 72)	—	—	3 (48, 52, 53)
	TCIA	1 (45)	—	—	—
	GDSC		—	—	1 (52)
	GEO	3 (44, 47, 49)	—	—	2 (52, 53)
	SEER	4 (23, 26, 30, 32)	—	—	—
	SMC	1 (27)	—	—	—
	AMC	1 (27)	—	—	—
Data types	Clinical	12 (23, 26, 27, 30, 32, 33, 45, 47, 50, 54, 71, 72)	3 (24, 37, 39)	6 (25, 28, 29, 40–42)	3 (43, 48, 53)
	WSI	1 (51)	1 (38)	—	1 (48)
	Ultrasound	—	1 (24)	—	—
	CT	2 (45, 50)	2 (37, 39)	1 (28)	1 (43)
	MRI	—	—	2 (25, 40)	—
	Molecular	7 (31, 45, 47, 49, 51, 54, 55)	1 (38)	—	1 (53)
Models	RF	5 (23, 30, 47, 71)	3 (24, 37, 39)	2 (29, 42)	3 (43, 52, 53)
	DT	4 (23, 30, 33)	—	1 (29)	1 (52)
	KNN	2 (23)	1 (24)	1 (29)	2 (43, 52)
	LR	7 (26, 30, 44, 45, 47, 55, 71)	2 (24, 37)	1 (29)	2 (43, 52)
	SVM	2 (23)	1 (37)	3 (29, 40, 42)	2 (43, 52)
	XGBoost	5 (23, 44, 47, 71)	1 (37)	1 (29)	2 (43, 52)
	AdaBoost	2 (23)	—	—	—
	DL	5 (32, 50, 51, 54, 72)	1 (38)	3 (25, 28, 40)	1 (48)
	Others	7 (27, 30, 31, 34, 49, 71)	—	2 (41, 42)	2 (43, 52)
Evaluations	Accuracy	7 (23, 30, 32, 34, 44, 55, 71)	1 (24)	2 (29, 42)	1 (52)
	Sensitivity	5 (23, 34, 44, 55, 71)	1 (24)	1 (29)	—
	Specificity	4 (23, 34, 44, 71)	—	1 (29)	—
	Precision	2 (30, 55)	1 (24)	—	—
	AUC	10 (23, 27, 30, 33, 34, 45, 47, 50, 71, 72)	4 (24, 37–39)	3 (25, 29, 40)	1 (43)
	C‐index	6 (26, 31, 49–51, 54)	—	4 (25, 28, 40, 41)	1 (53)
	Others	2 (23, 44)	2 (24, 38)	—	1 (43)

### 3.2 Datasets Characteristics

The datasets utilized in the reviewed studies were categorized into open‐ and closed‐access datasets. Open‐access datasets were utilized in 16 studies, all focusing on OS outcomes. Among these, 10 studies relied on datasets from The Cancer Genome Atlas (TCGA), five from the Gene Expression Omnibus (GEO), and four from the Surveillance, Epidemiology, and End Results (SEER) datasets. The SEER database provided the largest dataset, containing 42 827 records [33], whereas the smallest dataset included clinical data and ultrasound images for 64 patients [26]. Nine studies utilized datasets containing over 1000 samples [24, 25, 33, 41, 42, 45, 46, 49, 51], with six studies concentrating on OS using tabular data [24, 33, 41, 42, 46, 49] and two focusing on RFS using imaging data [25, 45]. Fifteen studies utilized closed‐access datasets sourced from healthcare institutions. Among these, five studies focused on OS [27, 32, 35, 36, 41], four on PFS [26, 37, 38, 50], six on RFS [23, 25, 30, 44, 45, 51], and one on TRP [52].

### 3.3 Data Cleaning and Preprocessing

Data wrangling was performed across all studies, though detailed descriptions of these processes were often sparse. Common data cleaning steps included the removal of duplicate records, correction of structural errors, addressing missing data, and identification of outliers [27, 28, 33]. Statistical methods and ML models were commonly utilized to manage missing data. For instance, some studies employed imputation methods using the mean, median, or mode [27], whereas others applied ML models such as *K*‐nearest neighbor (KNN) [24, 27], Elastic Net algorithm [43], multiple chained equations [36], and random forest (RF) [50]. In one study, features with over 50% missing data were excluded from analyses [27]. Feature selection was predominantly carried out using supervised and unsupervised ML algorithms, including Fisher's exact test [55], recursive feature elimination (RFE) [26], LASSO [30, 34, 43, 50], convolutional neural networks (CNNs) [25, 37, 38, 45], RF [34], graph neural network (GNN) [53], and XGBoost [26, 28, 34]. Prior to modeling, the distribution and independence of features were assessed using various statistical tests, including *T*‐tests, Wilcoxon, Mann–Whitney, McNemar, the chi‐squared test, Pearson, and Spearman correlations [24, 26, 29, 33, 34, 35]. One study [32] utilized the National Comprehensive Cancer Network (NCCN) guidelines to inform feature selection.

### 3.4 Modeling

The most frequently used models for survival prediction were RF, support vector machine (SVM), logistic regression (LR), XGBoost, and DL algorithms. RF and XGBoost were the predominant models applied to OS prediction, whereas RF and LR were common in PFS prediction, and DL was primarily used in RFS prediction. Although most included studies employed multiple ML models for OC survival prediction, some studies opted for simpler ML models, largely due to the reliance on traditional statistical methods for prognosis.

Innovative ML models were explored in several studies for OC prognosis, including vision transformers (ViTs) [35], bagging‐based models [28], attention‐based learning [37, 40], graph‐based learning [53], and fusion models [41, 45, 46]. Among studies comparing multiple models, XGBoost achieved the best performance in four studies [28, 31, 38, 51], followed by RF [26, 33, 54]. Other models, such as bagging [27], gradient boosting [24], CatBoost [52], and LR [31, 42], each demonstrated superior performance in individual studies. Cross‐validation was the most commonly used method for training and validating ML models [26, 27, 28, 29, 31, 32, 37, 38, 50], whereas a few studies employed leave‐one‐out validation [30, 55]. In some studies [25, 26, 30, 38, 45, 53], DL models were used as feature extractors, with ML algorithms subsequently applied for survival prediction.

### 3.5 Model Optimization and Evaluation

Meta‐parameter tuning was employed in some studies to optimize ML models, using methods such as grid search, random search, and Bayesian optimization [36, 37, 47]. Regularization, batch normalization, and learning rate scheduling were also applied in several studies to optimize DL models [25, 37, 38]. Evaluation methods varied significantly, with nine studies implementing external validation [24, 25, 28, 29, 34, 38, 52, 53, 54], whereas the remaining studies relied on internal validation. Among evaluation metrics, the area under the curve (AUC) was the most frequently reported, appearing in 18 studies, followed by accuracy in 11 studies, sensitivity in seven studies, and the C‐index in 11 studies (Table 3). Studies often allocated between 10% and 30% of their data for ML model evaluation [31, 32, 43, 45, 47]. The maximum and minimum outputs of ML models based on each evaluation criterion were compiled (Table 4).

**TABLE 4:** Classification of evaluation criteria used based on the type of survival (from the lowest to the highest).

Evaluation criteria	OS		PFS		RFS		TRP	
	Min	Max	Min	Max	Min	Max	Min	Max
AUC	0.64 (55)	0.94 (72)	0.62 (37)	0.92 (24)	0.78 (29)	0.98 (25)	0.87 (43)	0.87 (43)
Accuracy	0.58 (44)	0.95 (32)	0.93 (24)	0.93 (24)	0.80 (29)	0.90 (42)	0.82 (52)	0.82 (52)
Sensitivity	0.34 (55)	0.82 (44)	0.90 (24)	0.90 (24)	0.73 (29)	0.73 (29)	—	—
Specificity	0.38 (44)	0.92 (23)	—	—	0.71 (29)	0.71 (29)	—	—
C‐index	0.73 (54)	0.79 (31)	—	—	0.62 (40)	0.75 (25)	0.75 (53)	0.75 (53)

### 3.6 Influential Variables

Most of the included studies identified key clinical variables that significantly influenced the prediction of OC survival. Among these, age at diagnosis, tumor stage, histology, tumor grade, chemotherapy, metastasis status, race, CA‐125 levels, lymph node involvement, surgery information, and tumor diameters consistently emerged as critical determinants. Six studies also integrated molecular data, identifying factors such as BRCA1, NBN, BRIP1, RAD50, PTEN, and PMS2, which positively correlated with survival, whereas FANCE, FOXM1, KRAS, FANCD2, TTN, and CSMD3 were associated with poorer outcomes [48]. Additional influential variables included CCR5 expression levels [43], CT–based radiomics [43], and serum proteomics [44]. Furthermore, health‐related quality of life and psychosocial factors were highlighted in several studies as important contributors to survival outcomes [36]. Collectively, these variables provide a comprehensive framework for improving prognostic accuracy in OC (Table 5).

**TABLE 5:** Significant variables in ovarian cancer survival prediction.

Important variables	OS	PFS	RFS	TRP
Age at diagnosis	9 (23, 26, 27, 30, 33, 50, 51, 54, 72)	1 (24)	1 (42)	—
Tumor stage	5 (23, 26, 27, 50, 54)	1 (39)	2 (28, 42)	—
Histology	3 (23, 26, 27)	—	—	—
Chemotherapy	3 (23, 30, 71)	1 (24)	—	—
Tumor grade	4 (23, 26, 27, 51)	—	—	—
Metastasis	—	1 (24)	—	—
Race	3 (23, 26, 54)	—	—	—
CA‐125 levels	3 (27, 33, 50)	2 (39, 41)	2 (28, 41)	—
Lymph node status	—	1 (24)	—	—
Surgery information	1 (27)	—	—	—
Tumor diameters	2 (45, 50)	—	—	—
Others	11 (23, 27, 30, 31, 33, 34, 45, 54, 55, 71, 72)	2 (24, 39)	—	3 (43, 52, 53)

## 4 Discussion

This systematic review comprehensively analyzed 2400 articles across four databases, leading to the final inclusion of 32 original studies. These studies addressed OC prognosis, utilizing ML and DL models as predictive tools. The datasets utilized in these studies ranged significantly in size and type, from small collections to large‐scale datasets, and included tabular, image‐based, and molecular data. Key clinical variables frequently identified as influential in these studies include age at diagnosis, tumor stage, histology, and treatment types.

While AI models demonstrate promising accuracy compared to non‐AI methods for survival prediction, this potential can be misleading without rigorous validation and use of heterogeneous, real‐world datasets. The reliance on homogeneous, single‐institution datasets in some studies limits generalizability, highlighting the challenges of translating AI–driven detailed predictions into clinical practice [56].

Compared to traditional statistical models, interpretable ML models offer enhanced transparency and understandability by revealing feature influence on predictions, fostering clinical trust [57]. Furthermore, ML's ability to handle complex, large datasets and focus on individual patient characteristics, rather than population averages, enables more personalized survival predictions, highlighting their potential clinical utility [58].

Dataset selection is crucial for predictive model reliability and applicability; open‐access datasets, with larger samples and accessibility, enhance transparency, reproducibility, and collaboration. Conversely, small datasets pose overfitting risks, compromising generalizability, thus emphasizing careful dataset selection for advancing knowledge and scientific discovery [59, 60].

Data cleaning and preprocessing are fundamental for ensuring data quality, reducing errors, and mitigating bias, which are crucial for reliable predictive model training. Their methodological rigor directly impacts the validity and accuracy of survival prediction models [61, 62].

Imbalanced datasets, a frequent issue in medical research, are often addressed with techniques like SMOTE [32, 33] and cost‐sensitive [26] methods to reduce bias and enhance reliability. However, recent findings suggest these corrections may not improve predictive performance and can produce inaccurate probability estimates, highlighting the need for caution when applying such methods [63].

XGBoost and RF emerged as top‐performing ML algorithms in accuracy and interpretability, whereas decision trees remain favored in clinical settings for their simplicity and ease of implementation [64].

The integration of clinical, imaging, and molecular data significantly enhances the accuracy and reliability of ML models for OC survival prediction, with multidimensional approaches showing superior predictive performance. Image datasets, supported by advanced processing algorithms, often outperform tabular data, with CNNs traditionally used for medical image analysis despite their focus on local information [12, 65]. Transformer models, utilizing self‐attention mechanisms, overcome this limitation by aggregating global information and capturing broader spatial patterns [35]. Additionally, GNNs effectively model relational data, such as molecular networks and tissue interactions, whereas ViTs excel in analyzing imaging data [66]. Integrating these approaches presents a potentially valuable strategy for constructing comprehensive models capable of generating dependable predictive assessments.

In OC survival studies, feature selection and model interpretability are crucial for developing clinically relevant ML models. Identifying key features like age and tumor stage improves model accuracy. Techniques such as SHAP [67] and LIME [68] enhance transparency by explaining feature influence, fostering trust and facilitating integration into personalized medicine for improved clinical decisions.

A significant limitation in the current body of literature is the lack of reporting on study quality and the tendency to overlook potential biases. For instance, achieving an accuracy rate above 90% might superficially suggest that prognostication challenges have been resolved. However, this assumption is misleading due to persistent issues like overfitting, insufficient transparency and interpretability in model development, and a lack of robust external validation. AI research in OC often does not sufficiently address these critical limitations, which are vital for accurately assessing the true performance and clinical applicability of these models.

Advancing the field requires addressing several key areas to bring AI–based models closer to clinical utility. First and foremost, the implementation of rigorous validation protocols must become standard practice. External validation employing diverse, multi‐institutional datasets is crucial. These methods are critical for evaluating the robustness of models and their ability to generalize to new, unseen data. Additionally, reporting confidence intervals and employing statistical tests for model comparison will provide clinicians with more reliable insights into the relative performance of these models, enabling more informed decision‐making in clinical settings.

Another crucial consideration is the integration of AI models into the clinical workflow. These models should not be perceived as standalone solutions but rather as tools designed to assist clinicians, particularly in navigating complex decision‐making processes. Future research should prioritize understanding how these models can function within clinical environments, possibly serving as assistive tools that offer second opinions or illuminate novel insights that may be missed by human clinicians. This approach emphasizes the role of AI as a complementary resource rather than a replacement for clinical expertise [69].

Finally, enhancing the transparency and reproducibility of AI research is imperative. Researchers must adhere to standardized reporting guidelines, such as the TRIPOD checklist [70], to ensure that all relevant aspects of their models and datasets are thoroughly documented. Moreover, making code and datasets openly accessible, whenever possible, would significantly improve the reproducibility of research findings and allow for more rigorous testing of model generalizability. The current scarcity of openly accessible, heterogeneous datasets in OC research represents a major obstacle that must be overcome to facilitate more meaningful and clinically translatable AI advancements.

## 5 Conclusion

OC is a highly heterogeneous disease characterized by various histologic subtypes, making accurate prognosis particularly challenging. The application of ML has shown promise in addressing these complexities, particularly in managing missing data and integrating multiple predictive algorithms. This study identified RF, SVM, LR, XGBoost, and DL as the most frequently utilized models for survival prediction in OC.

Key predictive factors consistently identified across models included age at diagnosis, tumor stage, histologic subtype, treatment type, and specific biomarkers. The integration of diverse datasets—encompassing clinical, imaging, and molecular data—was found to enhance the accuracy of survival predictions. This suggests that combining heterogeneous, multidimensional data with advanced ML techniques offers a more robust approach to predicting OC survival. Moving forward, the emphasis should be on refining these models and exploring their potential for broader clinical application.

## Author Contributions

**Farkhondeh Asadi:** conceptualization, data curation, formal analysis, investigation, methodology, project administration, resources, supervision, validation, writing – original draft, writing – review and editing. **Milad Rahimi:** data curation, formal analysis, methodology, writing – original draft, writing – review and editing. **Nahid Ramezanghorbani:** writing – original draft, writing – review and editing. **Sohrab Almasi:** writing – original draft, writing – review and editing.

## Ethics Statement

This study was approved by Shahid Beheshti University of Medical Sciences (IR.SBMU.RETECH.REC.1401.660).

## Consent

The authors have nothing to report.

## Conflicts of Interest

The authors declare no conflicts of interest.

## Supporting information


**Data S1.** Supporting Information.

## Data Availability

All data used in the publication of this work were obtained from published studies. The abstracts for these studies are available in the PubMed, Scopus, Web of Science, and Cochrane database.

## References

[1] R. Nopour , “Screening Ovarian Cancer by Using Risk Factors: Machine Learning Assists,” BioMedical Engineering OnLine 23, no. 1 (2024): 18. 38347611 10.1186/s12938-024-01219-x PMC10863117

[2] F. Bray , M. Laversanne , H. Sung , et al., “Global Cancer Statistics 2022: GLOBOCAN Estimates of Incidence and Mortality Worldwide for 36 Cancers in 185 Countries,” CA: A Cancer Journal for Clinicians 74, no. 3 (2024): 229–263. 38572751 10.3322/caac.21834

[3] B. Shaik , T. Zafar , K. Balasubramanian , and S. P. Gupta , “An Overview of Ovarian Cancer: Molecular Processes Involved and Development of Target‐Based Chemotherapeutics,” Current Topics in Medicinal Chemistry 21, no. 4 (2021): 329–346. 33183204 10.2174/1568026620999201111155426

[4] G. C. Jayson , E. C. Kohn , H. C. Kitchener , and J. A. Ledermann , “Ovarian Cancer,” Lancet 384, no. 9951 (2014): 1376–1388. 24767708 10.1016/S0140-6736(13)62146-7

[5] S. Lheureux , C. Gourley , I. Vergote , and A. M. Oza , “Epithelial Ovarian Cancer,” Lancet 393, no. 10177 (2019): 1240–1253. 30910306 10.1016/S0140-6736(18)32552-2

[6] Z. Momenimovahed , A. Tiznobaik , S. Taheri , and H. Salehiniya , “Ovarian Cancer in the World: Epidemiology and Risk Factors,” International Journal of Women's Health 11 (2019): 287–299. 10.2147/IJWH.S197604 PMC6500433 31118829

[7] S. J. Ramus and S. A. Gayther , “The Contribution of BRCA1 and BRCA2 to Ovarian Cancer,” Molecular Oncology 3, no. 2 (2009): 138–150. 19383375 10.1016/j.molonc.2009.02.001 PMC5527889

[8] M. A. Vázquez , I. P. Mariño , O. Blyuss , et al., “A Quantitative Performance Study of Two Automatic Methods for the Diagnosis of Ovarian Cancer,” Biomedical Signal Processing and Control 46 (2018): 86–93. 30245736 10.1016/j.bspc.2018.07.001 PMC6146655

[9] L. C. Peres , K. L. Cushing‐Haugen , M. Köbel , et al., “Invasive Epithelial Ovarian Cancer Survival by Histotype and Disease Stage,” JNCI Journal of the National Cancer Institute 111, no. 1 (2019): 60–68. 29718305 10.1093/jnci/djy071 PMC6335112

[10] R. R. Hansebout , S. D. Cornacchi , T. Haines , and C. H. Goldsmith , “How to Use an Article About Prognosis,” Canadian Journal of Surgery 52, no. 4 (2009): 328. PMC2724829 19680521

[11] A. A. Wright , N. L. Keating , J. Z. Ayanian , et al., “Family Perspectives on Aaggressive Cancer Care Near the End of Life,” Journal of the American Medical Association 315, no. 3 (2016): 284–292. 26784776 10.1001/jama.2015.18604 PMC4919118

[12] C. Chen , T. W. Markossian , A. Silva , and Y. N. Tarasenko , “Epithelial Ovarian Cancer Mortality Among Hispanic Women: Sub‐Ethnic Disparities and Survival Trend Across Time: An Analysis of SEER 1992–2013,” Cancer Epidemiology 52 (2018): 134–141. 29306788 10.1016/j.canep.2017.12.003

[13] A. E. Stenzel , M. F. Buas , and K. B. Moysich , “Survival Disparities Among Racial/Ethnic Groups of Women With Ovarian Cancer: An Update on Data From the Surveillance, Epidemiology and End Results (SEER) Registry,” Cancer Epidemiology 62 (2019): 101580. 31400533 10.1016/j.canep.2019.101580

[14] M. Rutten , J. Boldingh , E. Schuit , et al., “Development and Internal Validation of a Prognostic Model for Survival After Debulking Surgery for Epithelial Ovarian Cancer,” Gynecologic Oncology 135, no. 1 (2014): 13–18. 25093289 10.1016/j.ygyno.2014.07.099

[15] L. A. Vale‐Silva and K. Rohr , “Long‐Term Cancer Survival Prediction Using Multimodal Deep Learning,” Scientific Reports 11, no. 1 (2021): 13505. 34188098 10.1038/s41598-021-92799-4 PMC8242026

[16] Y. L. Qiu , H. Zheng , A. Devos , H. Selby , and O. Gevaert , “A Meta‐Learning Approach for Genomic Survival Analysis,” Nature Communications 11, no. 1 (2020): 6350. 10.1038/s41467-020-20167-3 PMC7733508 33311484

[17] L. Vanneschi , A. Farinaccio , G. Mauri , M. Antoniotti , P. Provero , and M. Giacobini , “A Comparison of Machine Learning Techniques for Survival Prediction in Breast Cancer,” BioData Mining 4 (2011): 1–13. 21569330 10.1186/1756-0381-4-12 PMC3108919

[18] T. Ochi , K. Murase , T. Fujii , M. Kawamura , and J. Ikezoe , “Survival Prediction Using Artificial Neural Networks in Patients With Uterine Cervical Cancer Treated by Radiation Therapy Alone,” International Journal of Clinical Oncology 7, no. 5 (2002): 0294–0300. 10.1007/s101470200043 12402063

[19] J. Yao , S. Wang , X. Zhu , and J. Huang , eds., “Imaging Biomarker Discovery for Lung Cancer Survival Prediction,” in Medical Image Computing and Computer‐Assisted Intervention–MICCAI 2016: 19th International Conference, Athens, Greece, October 17–21, 2016, Proceedings, Part II 19 (Springer, 2016).

[20] S. Chen , L. Jiang , X. Zheng , et al., “Clinical Use of Machine Learning‐Based Pathomics Signature for Diagnosis and Survival Prediction of Bladder Cancer,” Cancer Science 112, no. 7 (2021): 2905–2914. 33931925 10.1111/cas.14927 PMC8253293

[21] A. Spooner , E. Chen , A. Sowmya , et al., “A Comparison of Machine Learning Methods for Survival Analysis of High‐Dimensional Clinical Data for Dementia Prediction,” Scientific Reports 10, no. 1 (2020): 20410. 33230128 10.1038/s41598-020-77220-w PMC7683682

[22] M. J. Page , J. E. McKenzie , P. M. Bossuyt , et al., “The PRISMA 2020 Statement: An Updated Guideline for Reporting Systematic Reviews,” International Journal of Surgery 88 (2021): 105906. 33789826 10.1016/j.ijsu.2021.105906

[23] C.‐J. Tseng , C.‐J. Lu , C.‐C. Chang , G.‐D. Chen , and C. Cheewakriangkrai , “Integration of Data Mining Classification Techniques and Ensemble Learning to Identify Risk Factors and Diagnose Ovarian Cancer Recurrence,” Artificial Intelligence in Medicine 78 (2017): 47–54. 28764872 10.1016/j.artmed.2017.06.003

[24] E. S. Paik , J.‐W. Lee , J.‐Y. Park , et al., “Prediction of Survival Outcomes in Patients With Epithelial Ovarian Cancer Using Machine Learning Methods,” Journal of Gynecologic Oncology 30, no. 4 (2019): e65. 31074247 10.3802/jgo.2019.30.e65 PMC6543110

[25] S. Wang , Z. Liu , Y. Rong , et al., “Deep Learning Provides a New Computed Tomography‐Based Prognostic Biomarker for Recurrence Prediction in High‐Grade Serous Ovarian Cancer,” Radiotherapy and Oncology 132 (2019): 171–177. 30392780 10.1016/j.radonc.2018.10.019

[26] F. Arezzo , G. Cormio , D. La Forgia , et al., “A Machine Learning Approach Applied to Gynecological Ultrasound to Predict Progression‐Free Survival in Ovarian Cancer patients,” Archives of Gynecology and Obstetrics 306 (2022): 1–12. 35532797 10.1007/s00404-022-06578-1 PMC9633520

[27] I. Kaur , M. Doja , T. Ahmad , et al., “An Integrated Approach for Cancer Survival Prediction Using Data Mining Techniques,” Computational Intelligence and Neuroscience 2021 (2021): 6342226. 34992648 10.1155/2021/6342226 PMC8727098

[28] Y.‐W. Hsiao , C.‐L. Tao , E. Y. Chuang , and T.‐P. Lu , “A Risk Prediction Model of Gene Signatures in Ovarian Cancer Through Bagging of GA‐XGBoost Models,” Journal of Advanced Research 30 (2021): 113–122. 34026291 10.1016/j.jare.2020.11.006 PMC8132202

[29] T. He , L. Huang , J. Li , P. Wang , and Z. Zhang , “Potential Prognostic Immune Biomarkers of Overall Survival in Ovarian Cancer Through Comprehensive Bioinformatics Analysis: A Novel Artificial Intelligence Survival Prediction System,” Frontiers in Medicine 8 (2021): 587496. 34109184 10.3389/fmed.2021.587496 PMC8180546

[30] H. M. Li , J. Gong , R. M. Li , et al., “Development of MRI‐Based Radiomics Model to Predict the Risk of Recurrence in Patients With Advanced High‐Grade Serous Ovarian Carcinoma,” American Journal of Roentgenology 217, no. 3 (2021): 664–675. 34259544 10.2214/AJR.20.23195

[31] N. B. Shannon , L. L. Y. Tan , Q. X. Tan , et al., “A Machine Learning Approach to Identify Predictive Molecular Markers for Cisplatin Chemosensitivity Following Surgical Resection in Ovarian Cancer,” Scientific Reports 11, no. 1 (2021): 1–10. 34413360 10.1038/s41598-021-96072-6 PMC8377048

[32] Y. Feng , Z. Wang , R. Cui , et al., “Clinical Analysis and Artificial Intelligence Survival Prediction of Serous Ovarian Cancer Based on Preoperative Circulating Leukocytes,” Journal of Ovarian Research 15, no. 1 (2022): 1–12. 35610701 10.1186/s13048-022-00994-2 PMC9129061

[33] A. Sorayaie Azar , S. Babaei Rikan , A. Naemi , et al., “Application of Machine Learning Techniques for Predicting Survival in Ovarian Cancer,” BMC Medical Informatics and Decision Making 22, no. 1 (2022): 345. 36585641 10.1186/s12911-022-02087-y PMC9801354

[34] Y. Li , Y. Nie , H. Guo , H. Guo , C. Ha , and Y. Li , “Establish of an Initial Platinum‐Resistance Predictor in High‐Grade Serous Ovarian Cancer Patients Regardless of Homologous Recombination Deficiency Status,” Frontiers in Oncology 12 (2022): 847085. 35372049 10.3389/fonc.2022.847085 PMC8971787

[35] Y. Zheng , F. Wang , W. Zhang , et al., “Preoperative CT‐Based Deep Learning Model for Predicting Overall Survival in Patients With High‐Grade Serous Ovarian Cancer,” Frontiers in Oncology 12 (2022): 986089. 36158664 10.3389/fonc.2022.986089 PMC9504666

[36] C. J. Sidey‐Gibbons , C. Sun , A. Schneider , et al., “Predicting 180‐Day Mortality for Women With Ovarian Cancer Using Machine Learning and Patient‐Reported Outcome Data,” Scientific Reports 12, no. 1 (2022): 21269. 36481644 10.1038/s41598-022-22614-1 PMC9732183

[37] C. Nero , L. Boldrini , J. Lenkowicz , et al., “Deep‐Learning to Predict BRCA Mutation and Survival from Digital H&E Slides of Epithelial Ovarian Cancer,” International Journal of Molecular Sciences 23, no. 19 (2022): 11326. 36232628 10.3390/ijms231911326 PMC9570450

[38] G. Avesani , H. E. Tran , G. Cammarata , et al., “CT‐Based Radiomics and Deep Learning for BRCA Mutation and Progression‐Free Survival Prediction in Ovarian Cancer Using a Multicentric Dataset,” Cancers 14, no. 11 (2022): 2739. 35681720 10.3390/cancers14112739 PMC9179845

[39] T. Lang , M. Yang , Y. Xia , et al., “Development of a Molecular Feature‐Based Survival Prediction Model of Ovarian Cancer Using the Deep Neural Network,” Genes & Diseases 10, no. 4 (2023): 1190. 37397532 10.1016/j.gendis.2022.10.011 PMC10311109

[40] M. Wu , C. Zhu , J. Yang , et al., “Exploring Prognostic Indicators in the Pathological Images of Ovarian Cancer Based on a Deep Survival Network,” Frontiers in Genetics 13 (2023): 1069673. 36685892 10.3389/fgene.2022.1069673 PMC9846244

[41] Q. Huan , S. Cheng , H. F. Ma , M. Zhao , Y. Chen , and X. Yuan , “Machine Learning‐Derived Identification of Prognostic Signature for Improving Prognosis and Drug Response in Patients With Ovarian Cancer,” Journal of Cellular and Molecular Medicine 28, no. 1 (2024): e18021. 37994489 10.1111/jcmm.18021 PMC10805490

[42] X. Liu , S. Jin , and D. Zi , “Overall Survival Prediction Models for Gynecological Endometrioid Adenocarcinoma With Squamous Differentiation (GE‐ASqD) Using Machine‐Learning Algorithms,” Scientific Reports 13, no. 1 (2023): 8395. 37225749 10.1038/s41598-023-33748-1 PMC10209095

[43] S. Wan , T. Zhou , R. Che , et al., “CT‐Based Machine Learning Radiomics Predicts CCR5 Expression Level and Survival in Ovarian Cancer,” Journal of Ovarian Research 16, no. 1 (2023): 1. 36597144 10.1186/s13048-022-01089-8 PMC9809527

[44] D. P. Mysona , S. Purohit , K. P. Richardson , et al., “Ovarian Recurrence Risk Assessment Using Machine Learning, Clinical Information, and Serum Protein Levels to Predict Survival in High Grade Ovarian Cancer,” Scientific Reports 13, no. 1 (2023): 20933. 38016985 10.1038/s41598-023-47983-z PMC10684567

[45] L. Liu , H. Wan , L. Liu , et al., “Deep Learning Provides a New Magnetic Resonance Imaging‐Based Prognostic Biomarker for Recurrence Prediction in High‐Grade Serous Ovarian Cancer,” Diagnostics 13, no. 4 (2023): 748. 36832236 10.3390/diagnostics13040748 PMC9954966

[46] G. P. Ghantasala , K. Dilip , P. Vidyullatha , et al., “Enhanced Ovarian Cancer Survival Prediction Using Temporal Analysis and Graph Neural Networks,” BMC Medical Informatics and Decision Making 24, no. 1 (2024): 299. 39390514 10.1186/s12911-024-02665-2 PMC11468212

[47] L. Jiang , C. Xu , Y. Bai , et al., “Autosurv: Interpretable Deep Learning Framework for Cancer Survival Analysis Incorporating Clinical and Multi‐Omics Data,” npj Precision Oncology 8, no. 1 (2024): 4. 38182734 10.1038/s41698-023-00494-6 PMC10770412

[48] M. C. Ma , E. S. Lavi , G. Altwerger , Z. P. Lin , and E. S. Ratner , “Predictive Modeling of Gene Mutations for the Survival Outcomes of Epithelial Ovarian Cancer Patients,” PLoS One 19, no. 7 (2024): e0305273. 38976671 10.1371/journal.pone.0305273 PMC11230535

[49] Y. Huang , J. A. Rauh‐Hain , T. H. McCoy , et al., “Comparing Survival of Older Ovarian Cancer Patients Treated With Neoadjuvant Chemotherapy Versus Primary Cytoreductive Surgery: Reducing Bias Through Machine Learning,” Gynecologic Oncology 186 (2024): 9–16. 38554626 10.1016/j.ygyno.2024.03.016 PMC13449683

[50] L. Jian , X. Chen , P. Hu , et al., “Predicting Progression‐Free Survival in Patients with Epithelial Ovarian Cancer Using an Interpretable Random Forest Model,” Heliyon 10 (2024): e35344. 39166005 10.1016/j.heliyon.2024.e35344 PMC11334804

[51] L.‐R. Yang , M. Yang , L.‐L. Chen , et al., “Machine Learning for Epithelial Ovarian Cancer Platinum Resistance Recurrence Identification Using Routine Clinical Data,” Frontiers in Oncology 14 (2024): 1457294. 39582538 10.3389/fonc.2024.1457294 PMC11581972

[52] W.‐C. Lin , C.‐S. Weng , A.‐T. Ko , et al., “Interpretable Machine Learning Model Based on Clinical Factors for Predicting Muscle Radiodensity Loss After Treatment in Ovarian Cancer,” Supportive Care in Cancer 32, no. 8 (2024): 544. 39046568 10.1007/s00520-024-08757-z

[53] Z. Yang , Y. Zhang , L. Zhuo , et al., “Prediction of Prognosis and Treatment Response in Ovarian Cancer Patients From Histopathology Images Using Graph Deep Learning: A Multicenter Retrospective Study,” European Journal of Cancer 199 (2024): 113532. 38241820 10.1016/j.ejca.2024.113532

[54] Q. Lin , W. Ma , M. Xu , et al., “A Clinical Prognostic Model Related to T cells Based on Machine Learning for Predicting the Prognosis and Immune Response of Ovarian Cancer,” Heliyon 10, no. 17 (2024): e36898. 39296051 10.1016/j.heliyon.2024.e36898 PMC11409031

[55] S. Kim , T. Park , and M. Kon , “Cancer Survival Classification Using Integrated Data Sets and Intermediate Information,” Artificial Intelligence in Medicine 62, no. 1 (2014): 23–31. 24997860 10.1016/j.artmed.2014.06.003

[56] V. D. Karalis , “The Integration of Artificial Intelligence Into Clinical Practice,” Applied Biosciences 3, no. 1 (2024): 14–44.

[57] A. Moncada‐Torres , M. C. van Maaren , M. P. Hendriks , S. Siesling , and G. Geleijnse , “Explainable Machine Learning Can Outperform Cox Regression Predictions and Provide Insights in Breast Cancer Survival,” Scientific Reports 11, no. 1 (2021): 6968. 33772109 10.1038/s41598-021-86327-7 PMC7998037

[58] K. Kourou , T. P. Exarchos , K. P. Exarchos , M. V. Karamouzis , and D. I. Fotiadis , “Machine Learning Applications in Cancer Prognosis and Prediction,” Computational and Structural Biotechnology Journal 13 (2015): 8–17. 25750696 10.1016/j.csbj.2014.11.005 PMC4348437

[59] R. F. Wolff , K. G. Moons , R. D. Riley , et al., “PROBAST: A Tool to Assess the Risk of Bias and Applicability of Prediction Model Studies,” Annals of Internal Medicine 170, no. 1 (2019): 51–58. 30596875 10.7326/M18-1376

[60] R. D. Riley , J. Ensor , K. I. Snell , et al., “Calculating the Sample Size Required for Developing a Clinical Prediction Model,” BMJ 368 (2020): m441. 32188600 10.1136/bmj.m441

[61] K. Stöger , D. Schneeberger , P. Kieseberg , and A. Holzinger , “Legal Aspects of Data Cleansing in Medical AI,” Computer Law and Security Review 42 (2021): 105587.

[62] Y.‐H. Hu , W.‐C. Lin , C.‐F. Tsai , S.‐W. Ke , and C.‐W. Chen , “An Efficient Data Preprocessing Approach for Large Scale Medical Data Mining,” Technology and Health Care 23, no. 2 (2015): 153–160. 25515050 10.3233/THC-140887

[63] R. van den Goorbergh , M. van Smeden , D. Timmerman , and B. Van Calster , “The Harm of Class Imbalance Corrections for Risk Prediction Models: Illustration and Simulation Using Logistic Regression,” Journal of the American Medical Informatics Association 29, no. 9 (2022): 1525–1534. 35686364 10.1093/jamia/ocac093 PMC9382395

[64] S. Höppner , E. Stripling , B. Baesens , S. vanden Broucke , and T. Verdonck , “Profit Driven Decision Trees for Churn Prediction,” European Journal of Operational Research 284, no. 3 (2020): 920–933.

[65] A. Zhang , L. Xing , J. Zou , and J. C. Wu , “Shifting Machine Learning for Healthcare From Development to Deployment and From Models to Data,” Nature Biomedical Engineering 6, no. 12 (2022): 1330–1345. 10.1038/s41551-022-00898-y PMC12063568 35788685

[66] K. Sone , Y. Toyohara , A. Taguchi , et al., “Application of Artificial Intelligence in Gynecologic Malignancies: A Review,” Journal of Obstetrics and Gynaecology Research 47, no. 8 (2021): 2577–2585. 33973305 10.1111/jog.14818

[67] S. M. Lundberg and S.‐I. Lee , “A Unified Approach to Interpreting Model Predictions,” Advances in Neural Information Processing Systems 30 (2017).

[68] M. T. Ribeiro , S. Singh , and C. Guestrin , eds., “"Why Should I Trust You?" Explaining the Predictions of Any Classifier,” in Proceedings of the 22nd ACM SIGKDD International Conference on Knowledge Discovery and Data Mining (KDD '16) (New York, NY: Association for Computing Machinery, 2016), 1135–1144, 10.1145/2939672.2939778.

[69] J. Breen , K. Allen , K. Zucker , et al., “Artificial Intelligence in Ovarian Cancer Histopathology: A Systematic Review,” npj Precision Oncology 7, no. 1 (2023): 83. 37653025 10.1038/s41698-023-00432-6 PMC10471607

[70] G. S. Collins , J. B. Reitsma , D. G. Altman , and K. G. Moons , “Transparent Reporting of a Multivariable Prediction Model for Individual Prognosis or Diagnosis (TRIPOD) the TRIPOD Statement,” Circulation 131, no. 2 (2015): 211–219. 25561516 10.1161/CIRCULATIONAHA.114.014508 PMC4297220

